# Temperature Dependence in Heterogeneous Nucleation with Application to the Direct Determination of Cluster Energy on Nearly Molecular Scale

**DOI:** 10.1038/s41598-017-16692-9

**Published:** 2017-12-04

**Authors:** Robert L. McGraw, Paul M. Winkler, Paul E. Wagner

**Affiliations:** 1 Environmental, and Climate Sciences Department, Brookhaven National Laboratory, Upton, NY 11973 USA; 2 University of Vienna, Faculty of Physics, Vienna, Austria

## Abstract

A re-examination of measurements of heterogeneous nucleation of water vapor on silver nanoparticles is presented here using a model-free framework that derives the energy of critical cluster formation directly from measurements of nucleation probability. Temperature dependence is correlated with cluster stabilization by the nanoparticle seed and previously found cases of unusual increasing nucleation onset saturation ratio with increasing temperature are explained. A necessary condition for the unusual positive temperature dependence is identified, namely that the critical cluster be more stable, on a per molecule basis, than the bulk liquid to exhibit the effect. Temperature dependence is next examined in the classical Fletcher model, modified here to make the energy of cluster formation explicit in the model.  The contact angle used in the Fletcher model is identified as the *microscopic* contact angle, which can be directly obtained from heterogeneous nucleation experimental data by a recently developed analysis method. Here an equivalent condition, increasing contact angle with temperature, is found necessary for occurrence of unusual temperature dependence. Our findings have immediate applications to atmospheric particle formation and nanoparticle detection in condensation particle counters (CPCs).

## Introduction

Temperature dependent nucleation measurements are a direct source for quantitative information on the energies needed to form the molecular-sized critical clusters that determine nucleation rate. Early double-piston cloud chamber measurements of homogeneous nucleation from the vapor revealed a systematic failure of classical nucleation theory (CNT) to correctly capture temperature dependence and, thereby, cluster energy^1,2^. Subsequent development of theory and computational models led to the finding that a temperature-dependent modification to the CNT cluster energy, derived from the Kelvin relation, restored agreement between theory, simulation, and measurement^3–5^.

For the case of heterogeneous nucleation from vapor, new interactions come into play as molecular clusters of condensate are adsorbed on or otherwise stabilized by the seed, which itself may be a single large molecule or molecular cluster of a composition different from that of the condensing vapor. Temperature dependent measurements of heterogeneous nucleation rate, determined here from measurements of the nucleation probability for seed activation, enable a new and direct way to determine the energetics of interaction between cluster and seed. While for homogeneous nucleation a comparatively small correction to CNT was found sufficient to restore agreement with measurement, for heterogeneous nucleation even the sign of the temperature dependence can differ from the expectations of CNT^6^. Resolution of this puzzle and estimation of critical cluster energies and heats of wetting at near molecular scale are objectives of the present study. Winkler *et al*., in a recently developed analysis method were able to directly determine contact line properties including contact angle for several different particle sizes at fixed temperature^7^. Here we focus on the non-isothermal case and make direct determination of cluster energies and microscopic heats of wetting likewise from nucleation probability measurements, but over a temperature range.

### Measurements Summary

Measurements using the size analyzing nucleus counter (SANC) reveal that the critical vapor saturation ratio for onset of heterogeneous nucleation can either increase or decrease with increasing temperature^6,8^. The positive temperature dependence (increasing onset saturation ratio with increasing temperature) is opposite the behavior observed in homogeneous nucleation where a decreasing onset saturation ratio with increasing temperature is the rule. Kupc *et al*. measured the nucleation of water vapor on silver nanoparticles and found that the critical saturation ratio passes through a maximum at about 278 K, with lower (higher) temperatures exhibiting the positive (negative) temperature dependence^8^.

**Figure 1:**
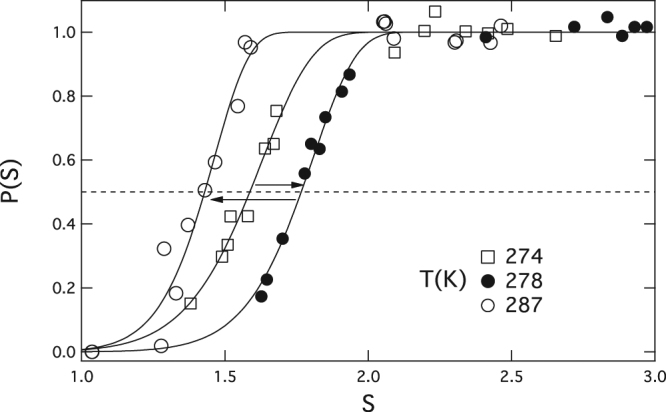
Nucleation probability curves at three different temperatures for seed particle radius ${r}_{p}=3.35\,nm$. The horizontal dashed line marks the onset condition (*P* = 0.5). Arrows point to higher temperature and show both increasing and decreasing *S*_*onset*_ with increasing *T*. Data points from SANC measurements of Kupc *et al*.^8^ Curves are fits to the Gumbel distribution.

The SANC measures heterogeneous nucleation probability *P*(*S*) as a function of saturation ratio, *S*. *P*(*S*) is related to nucleation rate by Equation (1):

1 $$P(S)=1-\frac{N({t}_{res})}{N(0)}=1-\exp [-{J}_{1}(S){t}_{res}]$$

where *N(t*_*res*_) is the (*S*-dependent) number concentration of unactivated seed particles remaining in the SANC at residence time *t*_*res*_, *N(0)* is the initial seed concentration and *J*_1_(*S*) (*s* ^−1^) is the heterogeneous nucleation rate per unactivated seed particle. For *S*_*onset*_ defined such that *P*(*S*_*onset*_) = 1/2, Eq. 2.1 gives ${J}_{1}({S}_{onset}){t}_{res}=\,\mathrm{ln}\,2$ and ${t}_{res}=\,\mathrm{ln}\,2/{J}_{1}({S}_{onset})$, thereby eliminating the SANC residence time from the calculations to follow. Applying the isothermal “first nucleation theorem” to *J*_1_ gives^9,10^

2 $${(\frac{\partial \mathrm{ln}{J}_{1}}{\partial \mathrm{ln}S})}_{T}={\rm{\Delta }}{n}^{\ast }+1$$

where ${\rm{\Delta }}{n}^{\ast }={n}^{\ast }-{n}_{0}$ is the excess number of condensate molecules present in the critical cluster, that is the number of molecules present in the critical cluster after subtracting off the number present in an equal volume of the parent phase, *n*_0_. For nucleation from vapor, the number of displaced molecules is small and to good approximation one can ignore the subtraction to obtain ${\rm{\Delta }}{n}^{\ast }={n}^{\ast }$ where n* is the actual number of molecules present in the critical cluster. Integration of Equation (2) neglecting higher-order terms (i.e. assuming constant n* over the *S*-range of the measurements at each temperature) and substituting into the exponent of Equation (1) yields an approximation for *P(S)* that describes the full range of measurements shown in Fig. 1 remarkably well^7^:

3 $$P(S)=1-\exp \{-\exp [{\rm{lnln}}2+({n}^{\ast }+1)(\mathrm{ln}\,S-\,\mathrm{ln}\,{S}_{onset})]\}$$

Equation (3) describes a cumulative Gumbel-type extreme value distribution in the parameters *S*_*onset*_ and *n* ^*^. As an aside, the high quality of fit is suggestive of a fundamental connection between nucleation (itself a rare event) and the statistics of extreme values^11^.

Fitting the Gumbel distribution, Equation (3), to SANC nucleation probability measurements to determine *S*_*onset*_ and *n* ^*^ is described in [7]. For the present study we obtain the fits to P(S) over each of three constant temperature data sets (markers and corresponding fit curves in Fig. 1). Values for *S*_*onset*_ and *n* ^*^, listed in columns 2 and 4 of Table 1, were obtained from the fits using:

4 $$\begin{array}{ccc}{\mathtt{P}}({{\mathtt{S}}}_{{\mathtt{o}}{\mathtt{n}}{\mathtt{s}}{\mathtt{e}}{\mathtt{t}}}{\mathtt{)}} & = & 0.5\\ {(\frac{dP(S)}{dT})}_{S={S}_{onset}} & = & \frac{ln2}{2}\frac{({n}^{\ast }+1)}{{S}_{onset}}\end{array}$$

**Table 1:** Model-independent parameters. *S*_*onset*_ and n* are from fits to the Gumbel distributions shown in Fig. 1. The last column gives the energy of formation of the critical cluster from an equal number of molecules of bulk liquid, ${\rm{\Delta }}{E}_{f}^{hetero}$, using Equation (9). Uncertainty limits at $T=278K$ are from Ref.^7^.

*r*_*p* (nm) = 3.35_					
*T(K)*	*S*_*onset*_	ln *S*_*onset*_	n*	$\frac{{\boldsymbol{d}}\,{\boldsymbol{ln}}\,{{\boldsymbol{S}}}_{{\boldsymbol{o}}{\boldsymbol{n}}{\boldsymbol{s}}{\boldsymbol{e}}{\boldsymbol{t}}}}{{\boldsymbol{d}}{\boldsymbol{T}}}$	${\boldsymbol{\Delta }}{{\boldsymbol{E}}}_{{\boldsymbol{f}}}^{{\boldsymbol{h}}{\boldsymbol{e}}{\boldsymbol{t}}{\boldsymbol{e}}{\boldsymbol{r}}{\boldsymbol{o}}}$ (10^−20^ joule)
274	1.59	0.464	9.7	0.0423	−54.0
278	1.77 ± 0.014	0.571	12.8 ± 0.7	0.0112	−23.6
287	1.43	0.358	12.3	−0.0586	81.6

For temperature-independent SANC residence time, *t*_*res*_ of Equation (1), the horizontal dashed line in Fig. 1 passes through values of *S* (here equal to *S*_*onset*_) at constant per particle nucleation rate. Under these conditions $d\,\mathrm{ln}\,{S}_{onset}/dT$ (column 5 of Table 1) equals the partial derivative ${{\mathtt{(}}{\rm{\partial }}{\rm{l}}{\rm{n}}{\rm{S}}/{\rm{\partial }}{\rm{T}})}_{{{\rm{J}}}_{{\mathtt{1}}}}$. The total derivative (column 5) is interpolated from the measured values of $\mathrm{ln}\,{S}_{onset}$ and *T* listed in the table by differentiating the parabola determined by the three measurement points:

5 $$\mathrm{ln}\,{S}_{onset}(T)=-302.276+2.16752T-0.00387821{T}^{2}$$

As seen from the Table 1 and Equation (5), *S*_*onset*_ can either increase or decrease with temperature and has a maximum value near 278 K. This interesting finding is analyzed in the following section.

## Results

The following sub-section presents a molecular-based criterion for the observed temperature dependence as derived from general physicochemical principles independent of any specific model or conceptual picture of the critical cluster.

### Model-independent Analysis

By way of contrast, consider first the homogeneous nucleation case where there are no seed interactions to consider and a “second nucleation theorem” has been developed that provides a molecular bases for temperature dependence of nucleation rate^12^:

6 $${(\frac{\partial \mathrm{ln}{J}_{{\rm{\hom }}}}{\partial T})}_{S}=\frac{{E}_{{A}_{{g}^{\ast }}}-{g}^{\ast }{E}_{{A}_{1}}^{bulk}}{k{T}^{2}}+\frac{{E}_{{A}_{1}}-{E}_{{A}_{1}}^{bulk}}{k{T}^{2}} > 0.$$

${E}_{{A}_{{g}^{\ast }}}$ is the energy of the unsupported critical cluster, ${A}_{{g}^{\ast }}$, consisting of *g* ^*^ monomers of condensed vapor species and ${E}_{{A}_{1}}^{bulk}$ and ${E}_{{A}_{1}}$ are the energies per molecule of *A* in its bulk liquid and vapor states, respectively. *g* and g* are used here in place of *n* and n* to distinguish from the heterogeneous nucleation case. The numerator of the second term on the right is the positive energy needed to vaporize a single molecule from bulk liquid. The numerator of the lead term on the right is also positive as the energy of an unsupported cluster is always higher than that of the same number of molecules present in bulk liquid due to the surface energy of the cluster. Accordingly, the derivative itself is positive and *J*_*hom*_ always increases with increasing temperature at constant *S*.

Only minor modification of Ford’s result, Equation (6), is required for the second nucleation theorem to carry over to temperature dependence of the per-seed heterogeneous nucleation rate, *J*_1_. Consider assembly from *bulk liquid* of the critical complex $M{A}_{{n}^{\ast }}$ consisting of a single seed particle *M* and its associated critical cluster ${A}_{{n}^{\ast }}$ through the equilibrium $M+{n}^{\ast }{A}_{1}\iff M{A}_{{n}^{\ast }}$. The extension of Equation (6) to this case is:

7 $${(\frac{\partial \mathrm{ln}{J}_{1}}{\partial T})}_{S}=\frac{{E}_{M{A}_{{n}^{\ast }}}-{n}^{\ast }{E}_{{A}_{1}}^{bulk}-{E}_{M}}{k{T}^{2}}+\frac{{E}_{{A}_{1}}-{E}_{{A}_{1}}^{bulk}}{k{T}^{2}}$$

which, as will be shown, can now have either sign. A simplified derivation is given in Methods. Equations (6) and (7) are independent of any specific model for the critical cluster e.g. independent of the spherical drop/cap models of CNT. These expressions are even independent of the classical separation of cluster free energy into surface and bulk components^12,13^.

To make contact with Fig. 1 we examine how *S*_*onset*_ varies with temperature, which as shown previously is equivalent to the variation of *S* with temperature at the constant per particle nucleation rate ${J}_{1}={J}_{1}({S}_{onset})$. At constant *J*_1_:

8a $$d\,\mathrm{ln}\,{J}_{1}={(\partial \mathrm{ln}{J}_{1}/\partial T)}_{lnS}dT+{(\partial \mathrm{ln}{J}_{1}/\partial \mathrm{ln}S)}_{T}d\,\mathrm{ln}\,S=0$$

and rearrangement of the second equality gives:

8b $${(\partial \mathrm{ln}S/\partial T)}_{\mathrm{ln}{J}_{1}}=-{(\partial \mathrm{ln}{J}_{1}/\partial T)}_{\mathrm{ln}S}/{(\partial \mathrm{ln}{J}_{1}/\partial \mathrm{ln}S)}_{T}.$$

The denominator on the right is generally positive, because an increasing saturation ratio at fixed *T* tends to lower the nucleation barrier and increase nucleation rate (cf. Equation (2)); the remaining partial derivatives then have opposite sign. (Exceptions to this rule exist but are not relevant to the vapor-to-liquid nucleation focus of the present study. For example, a negative denominator in the rhs of Equation (8b) occurs during efflorescence where water is a product of, rather than a reagent for, the nucleation step - after which only a dry salt crystal remains. Δ*n* ^*^ is negative for this case, which is why decreasing *S* favors the nucleation of efflorescence in a supersaturated salt-water solution droplet^14^). The usual temperature dependence, cf. Equation (6), for the homogeneous nucleation case, has ${(\partial \mathrm{ln}S/\partial T)}_{\mathrm{ln}{J}_{{\rm{\hom }}}} < 0$ and ${(\partial \mathrm{ln}{J}_{{\rm{\hom }}}/\partial T)}_{\mathrm{ln}S} > 0$. In the heterogeneous case both inequalities may flip direction depending on the sign of ${(\partial \mathrm{ln}{J}_{1}/\partial T)}_{\mathrm{ln}S}$. Combining the first and second nucleation theorems, Equations (2), (7) and (8), gives the temperature dependence of lnS at constant ${J}_{1}={J}_{1}({S}_{onset})$:

9 $$\frac{d\,\mathrm{ln}\,{S}_{onset}}{dT}=-\frac{({E}_{M{A}_{{n}^{\ast }}}-{n}^{\ast }{E}_{{A}_{1}}^{bulk}-{E}_{M})+({E}_{{A}_{1}}-{E}_{{A}_{1}}^{bulk})}{({n}^{\ast }+1)k{T}^{2}}.$$

The result can again have either sign depending on the sign and magnitude of the excess energy required to form the seed-associated critical cluster from n* molecules of bulk liquid, ${\rm{\Delta }}{E}_{f}^{hetero}={E}_{M{A}_{{n}^{\ast }}}-{n}^{\ast }{E}_{{A}_{1}}^{bulk}-{E}_{M}$. These formation energies, computed from Equation (9) using the parabola fit (Equation 5) to estimate $d\,\mathrm{ln}\,{S}_{onset}/dT$, are listed in the last column of Table 1. For strongly attractive seed-cluster interaction the formation energy can be negative ${\rm{\Delta }}{E}_{f}^{hetero} < 0$, as seen at the lowest two temperatures in the table. In these cases the supported critical cluster, despite the positive surface energy of its liquid-vapor interface, is overall more stable than the same number of molecules present in the bulk liquid reference state. Inspection of Equation (9) shows that a negative ${\rm{\Delta }}{E}_{f}^{hetero}$ is necessary for the unusual positive temperature dependence: $d\,\mathrm{ln}\,{S}_{onset}/dT > 0$. The related inequality of opposite sign: ${(\partial \mathrm{ln}{J}_{1}/\partial T)}_{lnS} < 0$ is likewise unusual, c.f. Equations (7) and (8). Sufficiency requires still further reduction in ${\rm{\Delta }}{E}_{f}^{hetero}$ to overcome the additional positive energy needed to vaporize a monomer from its bulk liquid state, ${E}_{{A}_{1}}-{E}_{{A}_{1}}^{bulk}=L-kT$ where *L* is the per-molecule latent heat of vaporization. The value, $L=7.44\times {10}^{-20}$ joule, was derived from the vapor pressure parameters for water^15^ and used in calculation of the formation energies given in Table 1.

The appearance of bulk energy terms, beginning with Equation (7) for the heterogeneous nucleation case, at first seems unexpected from a problem involving vapor condensation to form molecular clusters. Reflection shows this is due to the saturation ratio being held constant in taking the temperature derivative of $\mathrm{ln}\,{J}_{1}$. The bulk energy terms enter through the saturated vapor concentration, ${n}_{1}^{eq}(T)$, that appears in $S={n}_{1}/{n}_{1}^{eq}$. Keeping, instead, the external vapor concentration *n*_1_ constant while taking the temperature derivative yields new analogues to Equations (6) and (7). Focusing on the heterogeneous case, a straightforward application of the chain rule gives:

10 $${(\frac{\partial \mathrm{ln}{J}_{1}}{\partial T})}_{{n}_{1}}=\frac{{E}_{M{A}_{{n}^{\ast }}}-{n}^{\ast }{E}_{{A}_{1}}-{E}_{M}}{k{T}^{2}} < 0$$

and the bulk terms have vanished! Equation (10) is derived in Methods using a physically direct route based on the Gibbs-Helmholtz relation. Because positive energy is required to separate the cluster nucleus from its seed and convert it to vapor, the numerator on the right hand side of Equation (10) is negative and *J*_1_ is strictly a decreasing function of temperature when the derivative is evaluated at constant ${n}_{1}$. Applying the chain rule as in Equation (8), but with ln*S* replaced by ln ${n}_{1}$ gives

11 $${(\partial \mathrm{ln}{n}_{1}/\partial T)}_{\mathrm{ln}{J}_{1}}=-{(\partial \mathrm{ln}{J}_{1}/\partial T)}_{\mathrm{ln}{n}_{1}}/{(\partial \mathrm{ln}{J}_{1}/\partial \mathrm{ln}{n}_{1})}_{T}$$

and a result analogous to Equation (9) except that the terms involving bulk liquid energy and vaporization from the bulk no longer appear:

12 $$\frac{d\,\mathrm{ln}\,{n}_{1}^{onset}}{dT}=-\frac{({E}_{M{A}_{{n}^{\ast }}}-{n}^{\ast }{E}_{{A}_{1}}-{E}_{M})}{({n}^{\ast }+1)k{T}^{2}} > 0.$$

In this case the formation energy ${\rm{\Delta }}{E}_{f}^{vap}={E}_{M{A}_{{n}^{\ast }}}-{n}^{\ast }{E}_{{A}_{1}}-{E}_{M}$ (now relative to the vapor state) is negative and the onset vapor number concentration, ${n}_{1}^{onset}$, increases uniformly with temperature.

**Figure 2:**
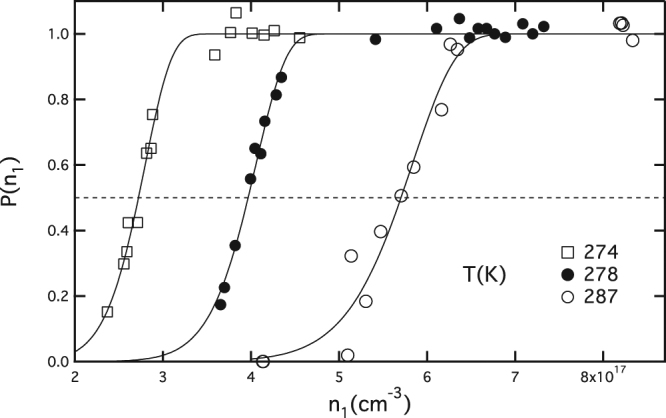
Same as Fig. 1 after units conversion from *S* to *n*_1_ (cm^−3^). In passing from Fig. 1 to Fig. 2, the nucleation probability curves are shifted horizontally to the right by the factor ${n}_{1}^{eq}(T)$. Largest shift occurs at the highest temperature with the result that the probability curve at 287 K overtakes the two at lower temperatures to give the reordering seen in the figure.

To illustrate this last effect Fig. 2 shows the same dataset as in Fig. 1, but after a transformation of coordinates from *S* to *n*_1_. Unlike ${S}_{onset}$, ${n}_{1}^{onset}$ is seen to increase uniformly with increasing temperature. An analogous Gumbel form results for the nucleation probability distribution $P({n}_{1})$ in terms of *n*_1_ and its two parameters; ${n}_{1}^{onset}$, for which $P({n}_{1}^{onset})=1/2$, and *n* ^*^, which is of course unchanged.

Signs of the various temperature dependencies are summarized in Table 2. Entries follow Equations (6) and (7) for the rate derivatives evaluated at constant *S* and Equation (10) for the heterogeneous nucleation rate derivative at constant *n*_1_. The remaining columns show temperature dependence of vapor concentrations expressed in terms of *S* or *n*_1_ from Equations (9) and (12), respectively. Only for heterogeneous nucleation expressed in terms of *S* can the sign of the temperature dependence go either way.

**Table 2:** Summary of temperature dependencies for homogeneous and heterogeneous vapor to liquid nucleation. For homogeneous nucleation *J* = *J*_*hom*_. For heterogeneous nucleation *J* is equal to the per particle nucleation rate, *J*_1_. Signs for the unusual temperature dependences are indicated by parenthesis.

Sign of →	${(\frac{{\boldsymbol{\partial }}\mathrm{ln}{\boldsymbol{J}}}{{\boldsymbol{\partial }}{\boldsymbol{T}}})}_{{n}_{1}}$	${(\frac{{\boldsymbol{\partial }}\mathrm{ln}{{\boldsymbol{n}}}_{{\bf{1}}}}{{\boldsymbol{\partial }}{\boldsymbol{T}}})}_{{\boldsymbol{J}}}$	${(\frac{{\boldsymbol{\partial }}\mathrm{ln}{\boldsymbol{J}}}{{\boldsymbol{\partial }}{\boldsymbol{T}}})}_{{\boldsymbol{S}}}$	${(\frac{{\boldsymbol{\partial }}\mathrm{ln}{\boldsymbol{S}}}{{\boldsymbol{\partial }}{\boldsymbol{T}}})}_{{\boldsymbol{J}}}$
Homogeneous Nucleation	−	+	+	−
Heterogeneous Nucleation	−	+	+ (−)	− (+)

### Perspective from classical nucleation theory

**Figure 3:**
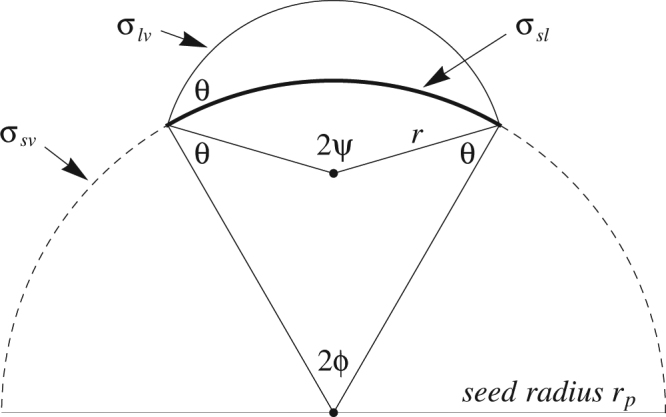
Spherical cap nucleus on an insoluble seed in the Fletcher model. Interfacial tensions and important angles: contact angle θ; polar angle ϕ, used in calculating the *sl* interfacial area; and ψ, used in calculating *lv* interfacial area, are indicated in the figure. The upper and lower points mark the centers of curvature of the spherical cap and the seed, respectively.

The preceding discussion has the advantage of being model independent, but may be too general for many applications. A more complete microphysical description of the interactions between the particle surface and the adsorbed critical cluster is needed to gain perspective on the energy scales involved. This section presents an analysis of the problem using Fletcher’s classical heterogeneous nucleation theory^16^. Hallmarks of the classical theory are that the condensate is modeled as an incompressible liquid, with phases assumed to retain uniform, bulk properties up to sharp (zero-volume) interfacial boundaries characterized by bulk (size independent) interfacial tensions. Without need for further assumptions, the Fletcher model is modified here to make contact with Equation (9). This requires making the energy of cluster formation explicit in the model. Temperature dependence of the contact angle (θ) between the solid-liquid and liquid-vapor interfaces, *sl* and *lv* (Fig. 3) is also explicit in the modification.

The Kelvin relation is used to derive the critical radius of curvature of the liquid-vapor surface of the spherical cap at saturation ratio *S* = *S*_*onset*_

13 $${r}^{\ast }=\frac{2{v}_{l}{\sigma }_{lv}}{kT\,\mathrm{ln}\,{S}_{onset}}$$

where *ν*_*l*_ is average volume per molecule in the bulk liquid phase. Remarkably, the contact angle *θ* can now be obtained directly from nucleation probability measurements for ${S}_{onset}$ and n*, the Kelvin relation for r*, and the known seed particle radius *r*_*p*_, as described in ref.^7^. The approach begins with the volume ratio, cap to spherical drop having the same radius of curvature r*^17^:

14 $$\begin{array}{c}{f}_{v}\equiv \frac{v\ast }{{v}_{dr}\ast }=\frac{1}{4}\{2+3(\frac{1-xm}{\sqrt{1+{x}^{2}-2xm}})-{(\frac{1-xm}{\sqrt{1+{x}^{2}-2xm}})}^{3}\\ \quad \quad \quad \quad \quad -{x}^{3}[2-3(\frac{x-m}{\sqrt{1+{x}^{2}-2xm}})+{(\frac{x-m}{\sqrt{1+{x}^{2}-2xm}})}^{3}]\}.\end{array}$$

${v}^{\ast }={n}^{\ast }{v}_{l}$ is cap volume, ${{v}_{dr}}^{\ast }=4\pi {({r}^{\ast })}^{3}/3$ is the volume of a spherical drop having the same radius of curvature ${r}^{\ast }$, ${f}_{v}$ is the volume ratio, and $x={r}_{p}/{r}^{\ast }$. As each of these quantities is known only $m=\,\cos \,\theta$ remaines to be determined. The contact angle in Table 3 is obtained from *m* as the positive real root of Equation (14).

**Table 3:** Continuation of Table 1 to include additional parameters derived using the Fletcher spherical cap model of classical nucleation theory. The critical radius, r*, results from measured *S*_*onset*_ and the Kelvin relation using surface tensions and density of water from ref.^15^. θ is the directly determined microscopic contact angle [7] and d cosΘ/dT its temperature dependence. Angle ψ, not tabulated, equals θ + ϕ (Fig. 3). ${{\rm{\Omega }}}_{sl}$ is the contact area between the cap and the seed and ${\rm{\Delta }}{H}_{w}$ is the microscopic heat of wetting from Equation (23). Uncertainty limits at $T=278K$ are from ref.^7^.

*r*_*p*_ (nm) = 3.35						
*T*(K)	r*(nm)	θ (deg)	ϕ (deg)	d cos θ/dT	Ω_*sl*_ (nm^2^)	Δ*H*_*w*_ (10^−20^ joule)
274	2.58	7.79	23.8	−0.0046	5.98	−125.
278	2.05	14.7 ± 0.3	20.8	−0.0030	4.61	−79.9
287	3.11	3.14	34.8	0.0029	12.6	−67.7

The reversible work required to fashion a spherical cap from bulk liquid is given by the surface component of Fletcher’s total free energy of cap formation^16^:

15 $${{\rm{\Phi }}}_{hetero}^{\ast }(\theta )={\sigma }_{lv}{{\rm{\Omega }}}_{lv}-({\sigma }_{sv}-{\sigma }_{sl}){{\rm{\Omega }}}_{sl}={\sigma }_{lv}{{\rm{\Omega }}}_{lv}-{\sigma }_{lv}\,\cos (\theta ){{\rm{\Omega }}}_{sl}$$

Ω_*lv*_ and Ω_*sl*_ are areas of the liquid-vapor and seed-liquid interfaces, respectively. ${\sigma }_{lv}$, ${\sigma }_{sv}$ and ${\sigma }_{sl}$ are interfacial tensions for the liquid-vapor, solid-vapor and solid-liquid interfaces, respectively. Young’s force balance equation:

16 $${\sigma }_{sv}-{\sigma }_{sl}={\sigma }_{lv}\,\cos (\theta ),$$

applied to the middle expression of Equation (15), uses the contact angle *θ* to determine the the interfacial tension difference ${\sigma }_{sv}-{\sigma }_{sl}$, a quantity not otherwise readily accessible by measurement, and one not required for calculation of *θ* from the measured nucleation parameters according to Equation (14). The interfacial areas appearing in Equation (15) are given by geometric formulae in terms of the critical radius, $r\ast$, seed particle radius ${r}_{p}$, and *θ*. With reference to Fig. 3 and the notation of Equation (14), these areas are^16^:

17a $${{\rm{\Omega }}}_{lv}=2\pi r{\ast }^{2}(1-\,\cos \,\psi )=2\pi r{\ast }^{2}(1+\frac{1-xm}{\sqrt{1+{x}^{2}-2xm}})$$

17b $${{\rm{\Omega }}}_{sl}=2\pi {r}_{p}^{2}(1-\,\cos \,\varphi )=2\pi {r}_{p}^{2}(1-\frac{x-m}{\sqrt{1+{x}^{2}-2xm}})$$

As previously mentioned, Equation (15) gives the *free energy* required to extrude the spherical cap from bulk liquid onto the seed, reducing to the unsupported spherical drop result of classical homogeneous nucleation theory for *θ* = 180°. The corresponding *energy* of formation (${\rm{\Delta }}{E}_{f}^{hetero}$ of Sec. 3) is obtained from the interfacial tensions (free energies) on replacement of $\sigma$ with $\sigma -T(d\sigma /dT)$ throughout, where the latter quantity is the energy per unit area of interface^18^. On applying this replacement and rearranging Equation (15), we obtain:

18 $$\begin{array}{c}{\rm{\Delta }}{E}_{f}^{hetero}\cong {{\rm{\Phi }}}_{hetero}^{\ast }-T\frac{d{{\rm{\Phi }}}_{hetero}^{\ast }}{dT}\\ \quad \quad \quad \,=\,{{\rm{\Omega }}}_{lv}({\sigma }_{lv}-T\frac{d{\sigma }_{lv}}{dT})+{{\rm{\Omega }}}_{sl}({\sigma }_{sl}-T\frac{d{\sigma }_{sl}}{dT})-{{\rm{\Omega }}}_{sl}({\sigma }_{sv}-T\frac{d{\sigma }_{sv}}{dT})\end{array}$$

where the approximate equality reflects the use of classical nucleation theory in the terms that follow.

The classical result, Equation (18), is now used together with the model independent Equation (9). Rearranging terms on the right hand side so as to apply Young’s equation, and combining with the model independent result gives:

19 $$\begin{array}{c}{\rm{\Delta }}{E}_{f}^{hetero}=-k{T}^{2}({n}^{\ast }+1)\frac{d\,\mathrm{ln}\,{S}_{onset}}{dT}-(L-kT)\cong \\ ({{\rm{\Omega }}}_{lv}-{{\rm{\Omega }}}_{sl}\,\cos \,\theta )({\sigma }_{lv}-T\frac{d{\sigma }_{lv}}{dT})+{{\rm{\Omega }}}_{sl}{\sigma }_{lv}T\frac{d\,\cos \,\theta }{dT}.\end{array}$$

The approximate equality again refers to use of CNT.

Equation (19) is used to evaluate $d\,\cos \,\theta /dT$ to obtain the results listed in Table 3; the other quantities appearing in this equation being known from tabulated thermo-physical properties^15^ and the measurement-inferred geometric quainties listed in Tables 1 and 3. For a convex substrate ${{\rm{\Omega }}}_{lv}\ge {{\rm{\Omega }}}_{sl}\ge {{\rm{\Omega }}}_{sl}\,\cos \,\theta$, and each of the terms in parenthesis following the approximate equality is positive, so the remaining term, containing $d\,\cos \,\theta /dT$, and this derivative itself, must be negative for ${\rm{\Delta }}{E}_{f}^{hetero} < 0$ and unusual temperature dependence: $d\,\mathrm{ln}\,{S}_{onset}/dT > 0$. In summary, we have shown from the modified Fletcher model that the condition $d\,\cos \,\theta /dT < 0$ (equivalently, $d\theta /dT > 0$) is necessary for unusual temperature dependence.

A similar analysis applies to the *entropy* of spherical cap formation from bulk liquid, ${\rm{\Delta }}{S}_{f}^{hetero}$. Replacing surface free energies $\sigma$ in Equation (15) with the corresponding surface entropies, $-d\sigma /dT$, and paralleling steps leading to Equation (19), yields:

20 $${\rm{\Delta }}{S}_{f}^{hetero}\cong -\frac{d{{\rm{\Phi }}}_{hetero}^{\ast }}{dT}={{\rm{\Omega }}}_{sl}{\sigma }_{lv}\frac{d\,\cos \,\theta }{dT}-({{\rm{\Omega }}}_{lv}-{{\rm{\Omega }}}_{sl}\,\cos \,\theta )\frac{d{\sigma }_{lv}}{dT}.$$

As surface tension decreases with increasing temperature, the last derivative is negative and the same necessary requirement for unusual temperature dependence,$d\,\cos \,\theta /dT < 0$ (equivalently $d\theta /dT > 0$), applies to have ${\rm{\Delta }}{S}_{f}^{hetero} < 0$ and a critical cap of lower entropy (more ordered) than the same amount of bulk liquid. Table 3 shows this condition is satisfied at temperatures at or below the maximum ${S}_{onset}$ temperature, 278 K.

**Figure 4:**
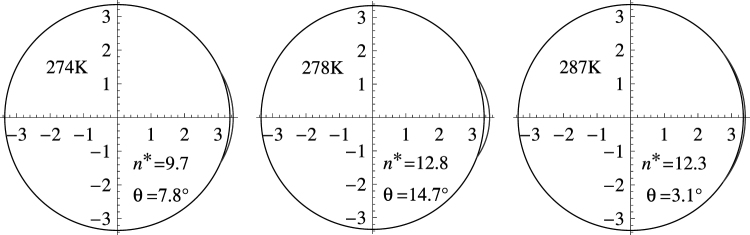
To-scale cross-sections of the critical seed-cap assembly consistent with the geometric factors listed in Table 3 (scale in nanometers).

Figure 4 shows to-scale cross sections of the critical seed-cap assembly constructed using the geometric parameters listed in Table 3. While the cap is just barely visible at the highest and lowest temperatures due to the small contact angles, implying nearly perfect wetting under these conditions, it is more prominent at the middle temperature, T = 278. From left to right in the figure one sees the clear increase in contact angle from 274 to 278 K and its decrease from 278 to 287 K; matching perfectly the increasing and decreasing temperature dependences observed in Fig. 1. This remarkable consistency with observations validates both the basic Fletcher model and its natural extension in this section to cap energy, entropy, and contact angle temperature dependence. Moreover, the necessary condition, $d\theta /dT > 0$, established here theoretically, is consistent with the empirical finding of Schobesberger *et al*. of being able to fit the Fletcher model to the unusual temperature dependence that they observed for n-propanol condensation on NaCl seed particles only by having the contact angle increase with increasing temperature^6^.

**Figure 5:**
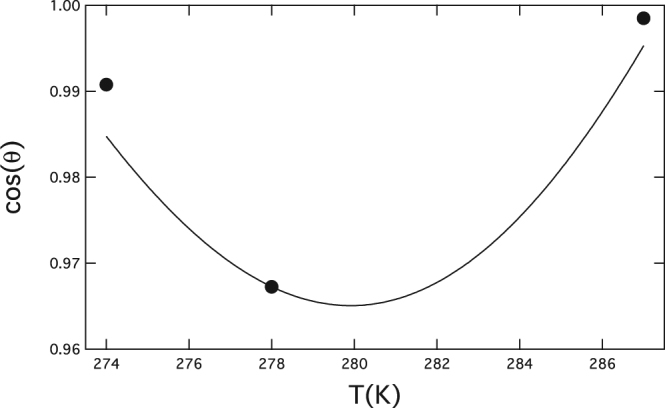
Markers: directly determined values of cos*θ* at the three temperatures as taken from the contact angles given in Table 3. Smooth curve: result of integrating Equation (19).

Equation 19 is a first order differential equation in *cosθ* that can be integrated numerically using Equation (5) to estimate $d\,\mathrm{ln}\,{S}_{onset}/dT$. On setting the boundary condition at the middle temperature $\theta (278K)={14.7}^{\circ }$, the smooth curve of Fig. 5 is obtained. The result, though limited by having measurements available at just three temperatures, shows consistency between the measurements-based determination of ${S}_{onset}$, the direct determination of contact angle, and Eq. 19 that combines the model-independent and CNT-based approaches.

### Direct determination of microscopic heats of wetting

For the condensed phase assembly of the critical complex from bulk liquid:

21 $$M+{n}^{\ast }{A}_{1}^{bulk}\iff M{A}_{{n}^{\ast }},$$

any exchange of volume work with the enviroment can generally be neglected making changes in energy and enthalphy essentially identical. In the classical theory, with its assumptions of zero-volume interfacial regions and incompressibility, the energy-enthalpy equality is exact.

An early suggestion by Harkins and Jura^19^ pointed out that the heat of wetting, consisting of the second and third terms on the right-hand-side of Equation (18):

22 $${\rm{\Delta }}{H}_{w}={{\rm{\Omega }}}_{sl}({\sigma }_{sl}-T\frac{d{\sigma }_{sl}}{dT})-{{\rm{\Omega }}}_{sv}({\sigma }_{sv}-T\frac{d{\sigma }_{sv}}{dT}),$$

should be obtainable directly by measuring temperature dependence of the contact angle. Neumann has reviewed such bulk measurements covering a number of substrate-liquid pairings using the method of capillary rise at a vertical plate^20^. Cases of both increasing and decreasing contact angle with temperature were observed and sign-changing slope discontinuities in *θ* vs. *T* were determined to accompany substrate phase changes that included changes in crystallinity and glass transitions as measured independently by differential thermal analysis (DTA) over the same temperature range.

Similar considerations, within the context of the Fletcher theory, apply on the microscopic scale to the contact angles and their temperature dependencies now determinable through nucleation probability measurements, thereby making microscopic heats of wetting directly accessible to measurement. Applying the Young relation to Equation (22) and rearranging terms gives:

23 $${\rm{\Delta }}{H}_{w}=-{{\rm{\Omega }}}_{sl}({\sigma }_{lv}-T\frac{d{\sigma }_{lv}}{dT})\cos \,\theta +{{\rm{\Omega }}}_{sv}{\sigma }_{lv}T\frac{d\,\cos \,\theta }{dT}$$

with the contact angle and interfacial areas taking on their microscopic values. Values of ${\rm{\Delta }}{H}_{w}$ calculated from Equation (23) are listed in Table 3. Negative values indicate exothermic wetting, with increasing exothermicity favored by having small contact angle, an increasing θ with increasing temperature, and large contact area - the same characteristics that favor negative ${\rm{\Delta }}{E}_{f}^{hetero}$ and unusual temperature dependence.

### Line tension

Given the remarkable consistency between predictions based on Fletcher’s model and the measurements of Kupc *et al*., it is incumbent to consider line tension, which Fletcher himself neglects writing^16^: “One new quantity becomes appreciable, however, for small embryos. This is the free energy associated with edges … we have to neglect its effects in the present discussion since no physical data are available”. This situation is now advanced by the ability to determine several microscopic properties, includimg contact angle, directly from measurement; providing some constraint on line tension through the generalized Young relation:

24 $$\cos \,(\theta )=\,\cos \,({\theta }_{Y})-\frac{{\kappa }_{g}\tau }{{\sigma }_{lv}}.$$

Here ${\theta }_{Y}$ is the bulk contact angle, $\tau$ is line tension and ${\kappa }_{g}$ is geodesic curvature of the three-phase contact line:

25 $${\kappa }_{g}=1/|{r}_{p}\,\tan \,\varphi |$$

(see ref.^7^ for a derivation of this quantity using differential geometry).

Line tension affect on heterogeneous nucleation manifest as corrections to the microscopic quantities appearing in Equations (15) and (16): *θ* and ${\sigma }_{sv}-{\sigma }_{sl}$ are no longer size independent as assumed in the Fletcher model. Lazaridis^21^ gives the correction to the surface work of nucleus formation as:

26 $$\begin{array}{c}{{\rm{\Phi }}}_{hetero}^{\ast }={\sigma }_{lv}{{\rm{\Omega }}}_{lv}-{\sigma }_{lv}\,\cos (\theta ){{\rm{\Omega }}}_{sl}-{\kappa }_{g}\tau {{\rm{\Omega }}}_{sl}+2\pi {r}_{p}\tau \,\sin \,\varphi \\ \quad \quad \,\,\,={\sigma }_{lv}{{\rm{\Omega }}}_{lv}-{\sigma }_{lv}\,\cos (\theta ){{\rm{\Omega }}}_{sl}+\kappa \tau {{\rm{\Omega }}}_{sl}\end{array}$$

in notation of Equation (15). The last equality, which appears to be new, uses the easily proven identity ${\kappa }_{g}{{\rm{\Omega }}}_{sl}+\kappa {{\rm{\Omega }}}_{sl}=2\pi {r}_{p}\,\sin \,\varphi =L$, where *L* is length of the three-phase contact line and $\kappa =1/({r}_{p}\,\sin \,\varphi )$ its Euclidean curvature, to reduced the line tension effect to a single term in ${{\rm{\Omega }}}_{sl}$ - essentially a size-dependent curvature correction to the adhesion tension ${\sigma }_{sv}-{\sigma }_{sl}$. This result makes clear that line tension cannot simply be folded into the microscopic contact angle $\theta$. It evidently has to remain an explicit and separate contribution to nucleation work.

**Figure 6:**
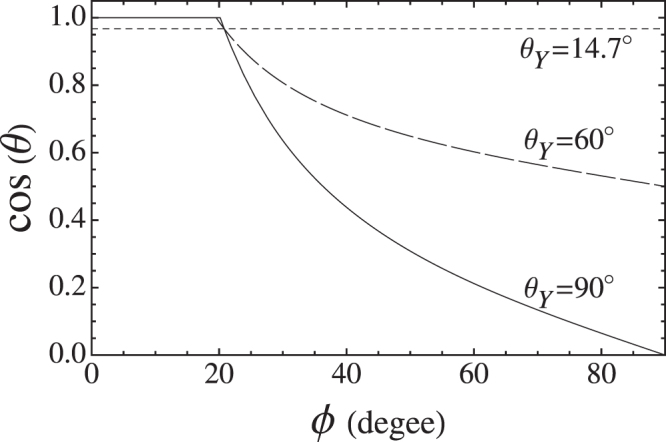
Cosine of the microscopic contact angle *θ* as a function of polar angle $\varphi$ for several values of ${\theta }_{Y}$, from Equations 24 and 25 with ${r}_{p}=3.35nm$. Constraint by the measured microscopic contact and polar angles, gives the crossing point {20.8, 0.967} for the angles from Table 3. Geodesic curvature vanishes at the seed equator (polar angle) where $\cos \,\theta =\,\cos \,{\theta }_{Y}=0.$

Equation (24) constrains $\tau$ as a function of ${\theta }_{Y}$ given that the contact and polar angles, *θ* and $\varphi$, are now directly determined by measurement (Fig. 6). A full determination of $\tau$ still requires independent measurement of ${\theta }_{Y}$ for a bulk surface representative of the microscopic surface of the seed. The line tension is estimated in ref.^7^ as ${\tau }_{90}=-0.92\times {10}^{-10}J/m$ (at 278 K) based on measurement of the bulk contact angle for water on silver, ${\theta }_{Y}={90}^{\circ }$ [ref.^22^]. In fact, the furnace-produced Ag-nanoparticles have an Ag-oxide surface. But it is notable in this connection that earlier measurements for oxidized and nonoxidized Ag-seed particles (surprisingly) did not result in any significant difference in heterogeneous nucleation behavior^22^.

Figure 6 has line tension negative over most of its range, increasing from ${\tau }_{90}=-0.92\times {10}^{-10}J/m$, for ${\theta }_{Y}={90}^{\circ }$, to zero for ${\theta }_{Y}$ equal to the microscopic contact angle, 14.7° (*ϕ*-independent dotted line). The figure also reveals a transition at $\varphi ={20.1}^{\circ }$, which value is not particularly sensitive to ${\theta }_{Y}$, below which there is wetting of the surface. Only for larger polar angles, in the “dry” region, can a spherical cap form^23^. Such wetting regions, expected to form spontaneously over the entire surface, would greatly enhance nucleation rate as does the negative contribution to surface energy from a negative $\tau$ in Eq. 26. It has even been suggested that negative line tension results in barrier-less heterogeneous nucleation without thermal activation^24^. This by itself would seem to preclude application of Fletcher’s theory, which is limited to activated barrier crossing. But other processes, such as conversion of an unstable wetting layer to a stable, molecularly dispersed adsorbed layer, could intervene. For example, the crossing point in Fig. 6 for curves having different ${\theta }_{Y}$ reflects the cap geometry, as layed out in Table 3. This point is close to the wetting transition and the cap might well be regarded as retaining contact properties of the wetting film which, in equilibrium, must have *positive* line tension as described by Rowlinson and Widom^23^ - otherwise the line between the wet and dry phases would spontaneously pucker to increase its length, so as to lower energy, and this lengthening would continue for as long as the line tension still had its negative value or until the wetting phase was molecularly dispersed^23^. Morever, a recent review makes reference to cases of rapid change from negative to positive line tension close to a wetting transition^25^. The preceding considerations could well be responsible for the success of Fletcher theory in our application.

## Conclusions

In this paper we demonstrated how the first and second nucleation theorems can be combined with measurements of nucleation probability at several different temperatures to provide a direct, model-free, determination of the energy of critical cluster formation. This determination provides quantitative measure of the strength of interaction between liquid condensate and nano-particle substrate at near molecular scale. Additionally, we presented a physical explanation for the unusual temperature dependence found in recent heterogeneous nucleation measurements and developed a quantitative cluster-energy based criterion for when this occurs. Finally, a complementary classical criterion for the effect was obtained by extending Fletcher’s classical heterogeneous nucleation model to explicitedly bring cluster energy and contact angle temperature dependence within its general framework.

As this paper was in final stage of preparation our attention was called to a set of temperature-dependent measurements of dielectric properties, differential heats of adsorption, and vapor pressures for water on Ag_2_O surface [Kuroda *et al*.^26^] that confirms many of the findings of the present study. Kuroda *et al*. interpreted their measurements by considering that a continuous phase transition occurs at around 278 K in the adsorbed layer, with a more ordered network structure, formed by hydrogen bonding between the adsorbed water molecules at lower temperatures. At higher temperatures, hydrophobic surface-cluster interactions were inferred from measured heats of adsorption that were less than the heat of liquefaction of water vapor. The Kuroda *et al*. interpretation is remarkably consistent with the critical cluster properties inferred from the present analysis of the Kupc *et al*.^8^ nucleation probability measurements: Similarities include proximity of a wetting transition at or around the same transition temperature, 278 K, as inferred in our case from the direct determination of critical cap geometry and analysis of line tension. Our finding of enthalpies of adsorption that are smaller (larger) than the enthalpy of bulk water liquefaction at temperatures higher (lower) than the transition temperature is supported by the Kuroda *et al*. vapor pressure measurements. Finally, the ordered state for adsorbed water below 278 K inferred by Kuroda *et al*. is fully consistent with our analysis of contact angle temperature dependence and its implication that the critical cap has lower entropy (is more ordered) than the same amount of bulk liquid in this region (Eq. 20).

## Methods

Here we obtain a simplified derivation of several of the key equations of Sec. 3 and trace their origin to fundamental physicochemical principles, namely the law of mass action and the Gibbs-Helmholtz relation. An approximation to the nucleation rate that includes the most important terms, while leaving out certain coefficients in the kinetic prefactor that are either small or will be eliminated anyway during the differentiation of logarithms that follows, uses the critical nucleus as transition state approximation:

27 $$J={n}_{M}{J}_{1}\cong {n}_{M}{n}_{1}{n}_{M{A}_{n\ast }}$$

Consider the following equilibria: (1) vaporization of $n\ast$ molecules from bulk liquid, $n\ast {A}_{1}^{bulk}\iff n\ast {A}_{1}$, (2) critical assembly from vapor, $M+n\ast {A}_{1}\iff M{A}_{n\ast }$, and (3) critical assembly from bulk liquid, $M+n\ast {A}_{1}^{bulk}\iff M{A}_{n\ast }$. The corresponding equilibrium constants are:

28 $${K}_{1}(T)={(\frac{[{A}_{1}]}{[{A}_{1}^{bulk}]})}^{n\ast }\cong {({n}_{1})}^{n\ast }$$

for vaporization,

29 $${K}_{2}(T)=\frac{[M{A}_{n\ast }]}{[M]{[{A}_{1}]}^{n\ast }}\cong \frac{{n}_{M{A}_{n\ast }}}{{n}_{M}{({n}_{1})}^{n\ast }}$$

for assembly from vapor, and ${K}_{3}(T)={K}_{1}{K}_{2}$ for assembly from bulk liquid. Activities are in square brackets and the activity of the bulk liquid standard state has been set to unity. Approximate equalities refer to use of the law of mass action to replace activities by concentrations - an excellent approximation for species present in the low-pressure vapor phase. Equation (10) follows immediately from Equations (27) and (29):

30 $${(\frac{\partial \mathrm{ln}{J}_{1}}{\partial T})}_{{n}_{1}}\cong {(\frac{\partial \mathrm{ln}{n}_{M{A}_{n\ast }}}{\partial T})}_{{n}_{1}}=\frac{d\,\mathrm{ln}\,{K}_{2}}{dT}=\frac{{E}_{M{A}_{g\ast }}-n\ast {E}_{{A}_{1}}-{E}_{M}}{k{T}^{2}}$$

where the last equality is the Gibbs-Helmholtz relation as it appears in terms of energies and vapor phase species number concentrations^13^ applied here to the heterogeneous nucleation case.

Equation (7), in which *S* is held constant, requires taking the temperature derivative of $\mathrm{ln}\,{n}_{1}=\,\mathrm{ln}\,S+\,\mathrm{ln}\,{n}_{1}^{eq}$ where ${n}_{1}^{eq}$ is the equilibrium vapor concentration. From Equation (28):

31 $$\frac{d\,\mathrm{ln}\,{K}_{1}}{dT}=n\ast \frac{d\,\mathrm{ln}\,{n}_{1}^{eq}}{dT}=n\ast \frac{L-kT}{k{T}^{2}}=n\ast \frac{{E}_{{A}_{1}}-{E}_{{A}_{1}}^{bulk}}{k{T}^{2}}$$

where *L* is enthalpy of vaporization. Finally, the combination of equations 27–31 gives Equation (7)

32 $$\begin{array}{rcl}{(\frac{\partial \mathrm{ln}{J}_{1}}{\partial T})}_{S} & \cong  & {(\frac{\partial \mathrm{ln}{n}_{1}{n}_{M{A}_{{n}^{\ast }}}}{\partial T})}_{S}={(\frac{\partial \mathrm{ln}{n}_{1}}{\partial T})}_{S}+\frac{d\,\mathrm{ln}\,{K}_{1}{K}_{2}}{dT}\\  & = & \frac{{E}_{M{A}_{{n}^{\ast }}}-{n}^{\ast }{E}_{{A}_{1}}-{E}_{M}}{k{T}^{2}}\\  & + & {n}^{\ast }\frac{{E}_{{A}_{1}}-{E}_{{A}_{1}}^{bulk}}{k{T}^{2}}+{n}^{\ast }\frac{{E}_{{A}_{1}}-{E}_{{A}_{1}}^{bulk}}{k{T}^{2}}\\  & = & \frac{{E}_{M{A}_{{n}^{\ast }}}-{n}^{\ast }{E}_{{A}_{1}}^{bulk}-{E}_{M}}{k{T}^{2}}+\frac{{E}_{{A}_{1}}-{E}_{{A}_{1}}^{bulk}}{k{T}^{2}}\end{array}$$

This derivation in particular highlights the distinction between whether *S* or ${n}_{1}$ is held constant while taking the temperature derivative. The distinction is revealed as having to do exclusively with whether the vaporization equilibrium (Eqs 28 and 31) needs or does not need, respectively, to be taken into account.

### Data availability

The present study uses only published data as cited.
